# Supplementary material for: Membrane-Interactive Compounds From Pistacia lentiscus L. Thwart Pseudomonas aeruginosa Virulence
Source: Front Microbiol. 2020 May 26;11:1068. doi: 10.3389/fmicb.2020.01068 (PMC7264755; doi:10.3389/fmicb.2020.01068)
Supplement: Supplementary file 1 [file Data_Sheet_1.PDF]

Supplementary Information

Membrane-Interactive Compounds from *Pistacia lentiscus* L. Thwart *Pseudomonas aeruginosa* Virulence

Ali Tahrioui[#,1], Sergio Ortiz[2], Onyedikachi Cecil Azuama[1], Emeline Bouffartigues[1], Nabiha Benalia[2], Damien Tortuel[1], Olivier Maillot[1], Smain Chemat[3], Marina Kritsanida[2], Marc Feuilloley[1], Nicole Orange[1], Sylvie Michel[2], Olivier Lesouhaitier[1], Pierre Cornelis[1], Raphaël Grougnet[2], Sabrina Boutefnouchet[2] & Sylvie Chevalier[1]

[1]Université de Rouen Normandie, Normandie Université, Laboratoire de Microbiologie Signaux et Microenvironnement, LMSM EA4312, Évreux, France

[2]Université Paris Descartes, Faculté des Sciences Pharmaceutiques et Biologiques, Équipe Produits Naturels, Analyses et Synthèses (PNAS), CiTCoM UMR 8038 CNRS, Paris, France

[3]Centre de Recherche Scientifique et Technique en Analyses Physico-Chimiques, CRAPC, Bou Ismaïl, Algérie

[#]Corresponding author:
Dr. Ali Tahrioui
Laboratory of Microbiology Signals and Microenvironment–LMSM EA4312, University of Rouen Normandy–Normandy University, 55 Rue Saint-Germain, 27000 Evreux, France
E-mail: ali.tahrioui@univ-rouen.fr
Phone: (+33) 2.32.29.15.60
Fax: (+33) 2.32.29.15.50

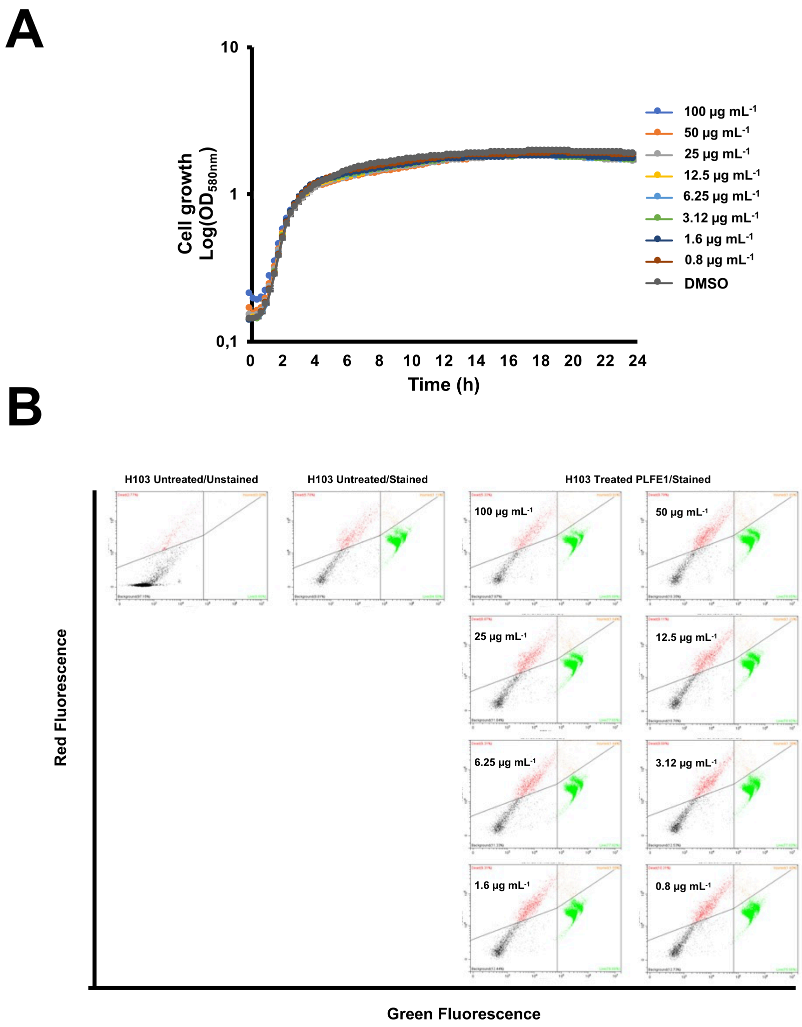

Figure S1. Effect of PLFE1 on *P. aeruginosa* growth and cell viability. (**A**) Growth kinetics of *P. aeruginosa* strain H103 treated with PLFE1 at various concentrations compared to the growth in the control condition (H103 untreated). Each point indicates the mean (± SEM) of OD_{580nm} values. (**B**) Flow cytometry analysis of H103 cell viability upon exposure to PLFE1 at various concentrations. Suspensions at a density of 1 x 10^6 CFU mL^{-1} were stained using the LIVE/DEAD™ BacLight™ Bacterial Viability and Counting Kit and then analyzed by flow cytometry. Live and dead cells emitting a green or a red fluorescence are indicated within a green or a red frame, respectively. The orange frame corresponds to cells with damaged membranes. The black frame corresponds to the background noise. Cytograms are representative of three independent experiments.

Table S1. Effect of PLFE1 on cell viability as determined by flow cytometry

**H103 cells**	**Normalized Live Events μL^{-1}**	**Normalized Injuried Events μL^{-1}**	**Normalized Dead Events μL^{-1}**
Untreated	0.963 ± 0.007	0.006 ± 0.001	0.029 ± 0.007
Treated with PLFE1 (μg mL^{-1})			
100	0.965 ± 0.006[a]	0.005 ± 0.001[a]	0.029 ± 0.007[a]
50	0.955 ± 0.003[a]	0.008 ± 0.002[a]	0.037 ± 0.002[a]
25	0.958 ± 0.001[a]	0.006 ± 0.001[a]	0.035 ± 0.002[a]
12.5	0.960 ± 0.002[a]	0.005 ± 0.000[a]	0.034 ± 0.002[a]
6.25	0.953 ± 0.006[a]	0.005 ± 0.001[a]	0.042 ± 0.007[a]
3.12	0.952 ± 0.002[a]	0.005 ± 0.001[a]	0.043 ± 0.002[a]
1.6	0.951 ± 0.002[a]	0.005 ± 0.001[a]	0.043 ± 0.002[a]
0.8	0.941 ± 0.006[a]	0.006 ± 0.001[a]	0.053 ± 0.006[a]

[a] Not Significant ($P \geq 0.05$)

A

B

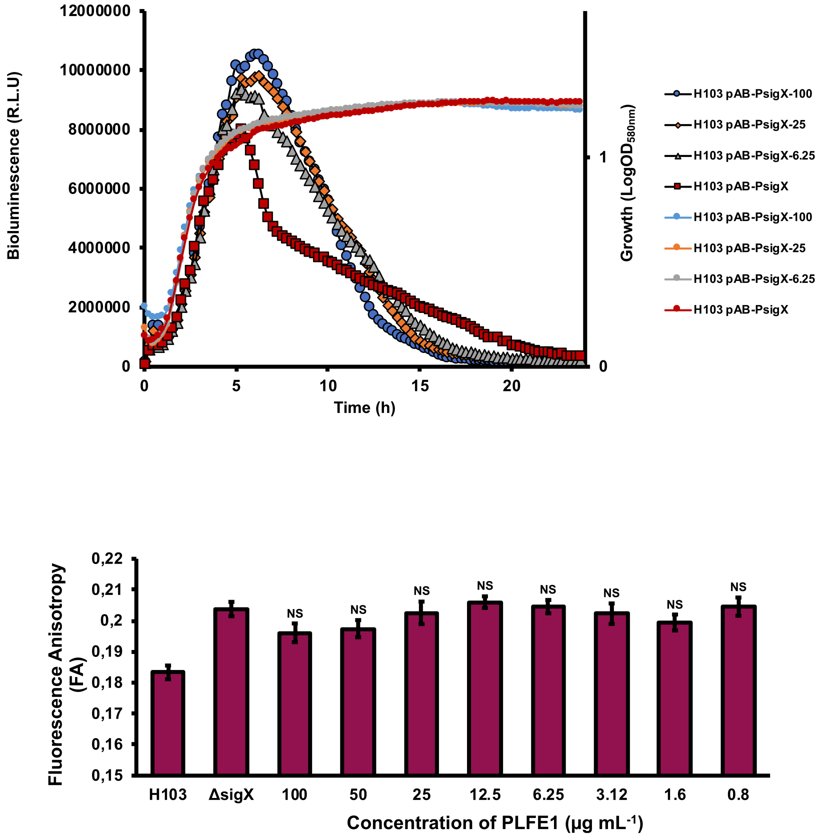

Figure S2. Effect of PLFE1 on the ECFσ SigX. (**A**) Relative bioluminescence levels of H103 strain harboring the pAB-P*sigX* plasmid (*sigX* promoter region) treated with PLFE1 at 100, 25, and 6.25 µg mL^{-1} compared to the relative bioluminescence levels in the control condition (H103 untreated). Growth kinetics are also displayed. (**B**) Fluorescence anisotropy (membrane fluidity) measurements in *P. aeruginosa* Δ*sigX* exposed to various concentrations of PLFE1 compared to the control condition (Δ*sigX* untreated). Values represent the mean (± SEM) of three independent assays. Statistics were achieved by a two-tailed *t* test: NS (Not Significant), $P \geq 0.05$.

Table S2. Fractions of PLFE1 obtained from MPLC

Code	Mass [mg]	Percentage [m/m, %]
PLFE1-1	3.0	1.5
PLFE1-2	35.5	17.8
PLFE1-3	16.5	8.2
PLFE1-4	16.2	8.1
PLFE1-5	16.8	8.4
PLFE1-6	9.6	4.8
PLFE1-7	4.2	2.1
PLFE1-8	21.3	10.6
PLFE1-9	5.3	2.6
PLFE1-10	1.2	0.6
PLFE1-11	8.7	4.4
PLFE1-12	6.1	3.0
PLFE1-13	2.9	1.4

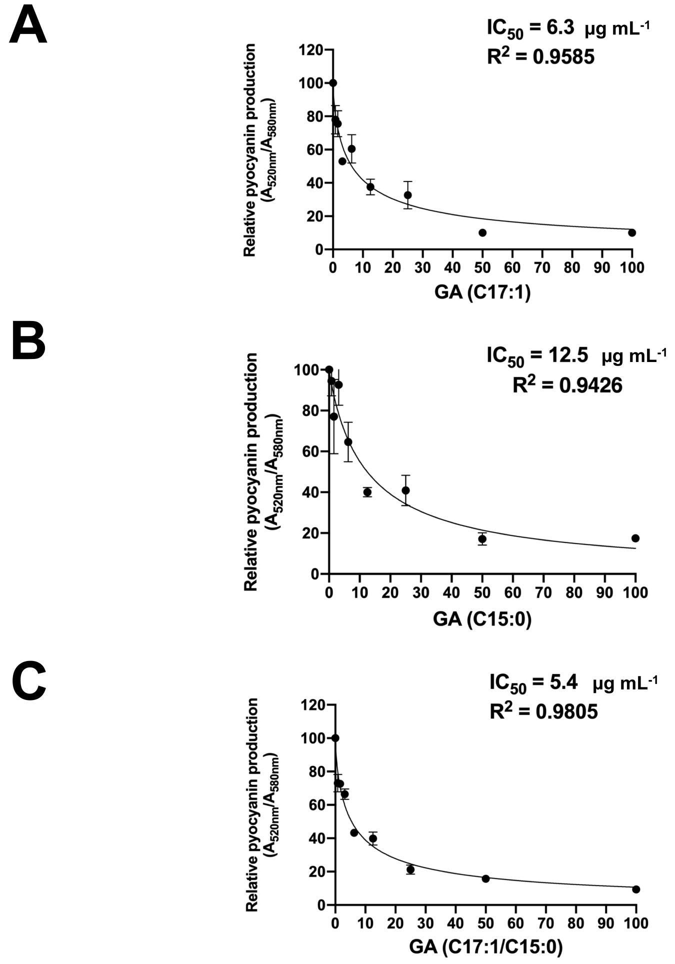

Figure S3. Determination of IC_{50} values for GA-enriched fractions. (**A**) GA (C17:1). (**B**) GA (C15:0) (**C**) mixture of GA (C17:1/C15:0).

Table S3. Collection and preparation of *P. lentiscus* L. fruit extracts

Plant material	Date of collection	Place	GPS position	Code	Solvent	Percent Yield
P. lentiscus L. fruit (PLF)	20/09/2016	Wilaya Jijel (Algeria)	36° 43' 25.248'' N 5° 52' 22.4148'' E	PLFE1	Cyclohexane	2.7
				PLFE2	Ethyl acetate	0.6
				PLFE3	Methanol	18.2
				PLFE4	Water	8.3

Table S4. List of primers used in this study

PA number	Gene name	Primer name	Sequence (5' > 3')[a]	Length
Construction of the transcriptional fusion pAB-P*sigX*				
PA1775-PA1776	*cmpX-sigX*	P*sigX*-<u>*SacI*</u>-F	taataa-**<u>GAGCTC-</u>**gagtcgctcggcctgca	29
		P*sigX*-<u>*SpeI*</u>-R	taataaa-**<u>CTAGTG-</u>**gtggaacagctccgagtgcg	33
Quantification of mRNA levels by RT-qPCR				
PA4210	*phzA*	*phzA-F*	AACCACTACATCCATTCCTTCG	22
		phzA-R	CGGCTATTCCCAATGCAC	18
PA0051	*phzH*	*phzH-F*	CGCGGGTTGGGTGGAT	16
		phzH-R	ATGACCGATACGCTCGCC	18
PA4209	*phzM*	*phzM-F*	GCTGCGCGTAATTTGATACAAG	22
		phzM-R	GATCCCGCTCTCGATCAGATC	21
PA4217	*phzS*	*phzS-F*	CCTGCGCGAATACGAAGAAG	20
		phzS-R	CGGCCCATTCCTCTTTTTC	19
PA0996	*pqsA*	*pqsA-F*	CGGAGTTGCTGGCATTGC	18
		pqsA-R	CTGTTGCCCATGCCATAGC	19
PA2587	*pqsH*	*pqsH-F*	CTCCATCGTGCAGATCCT	18
		pqsH-R	CGGAATGACGCAAGGTC	17
PA4190	*pqsL*	*pqsL-F*	CGGTATCGCCTCCTACGTG	19
		pqsL-R	GGAAGCTCACCACCAGTCG	19
PA1003	*pqsR*	*pqsR-F*	AACCTGGAAATCGACCTGTG	20
		pqsR-R	TGAAATCGTCGAGCAGTACG	20
PA1776	*sigX*	*sigX-F*	AATTGATGCGGCGTTACCA	19
		sigX-R	CCAGGTAGCGGGCACAGA	18
PA3639	*accA*	*accA-F*	TCTTCGGCAATCTGACCAGTT	21
		accA-R	GTAGCCGATGTAGTCGAGGGTA	22
PA4847	*accB*	*accB-F*	AAGCCATGAAGATGATGAACC	21
		accB-R	CGTTCTCCACCAGGATCGA	19
PA5174	*fabY*	*fabY-F*	AGGGCGACCTGGAGATCAT	19
		fabY-R	GCGCGTCCTTCTTGTATACCA	21
	16S	*16S-F*	AACCTGGGAACTGCATCCAA	20
		16S-R	CTTCGCCACTGGTGTTCCTT	20

[a]All the primers used in this study were synthesized by Eurogentec and are based on *P. aeruginosa* PAO1 genome sequence (http://www.pseudomonas.com). Bold nucleotides indicate restriction endonuclease sites inserted within primer sequences.
